# Evaluation of Changes in Equine Care and Limb-Related Abnormalities in Working Horses in Jaipur, India, as Part of a Two Year Participatory Intervention Study

**DOI:** 10.1371/journal.pone.0126160

**Published:** 2015-05-22

**Authors:** Helen R. Whay, Amit K. Dikshit, Jo Hockenhull, Richard M. A. Parker, Anindo Banerjee, Sue I. Hughes, Joy C. Pritchard, Christine E. Reix

**Affiliations:** 1 School of Veterinary Sciences, University of Bristol, Langford House, North Somerset, United Kingdom; 2 Help in Suffering, Maharani Farm, Durgapura, Jaipur, India; 3 Praxis Institute for Participatory Practices, C-75, South Extension, Part II, New Delhi, India; 4 Animals in International Development, Banwell, North Somerset, United Kingdom; 5 The Brooke, Friars Bridge Court, London, United Kingdom; The University of Melbourne, AUSTRALIA

## Abstract

**Background:**

Previous studies have found the prevalence of lameness in working horses to be 90–100%. Risk factors for lameness in this important equine population, together with risk-reduction strategies adopted by their owners, are poorly understood. The objective was to uncover risk factors for lameness and limb abnormalities in working horses, by associating clinical lameness examination findings on three occasions over two years with owner reported changes in equine management and work practices over this period.

**Methodology/Principal Findings:**

Twenty-one communities of horse owners in Jaipur, India, took part in a participatory intervention (PI) project aiming to reduce risk factors for poor welfare, particularly lameness and limb problems. Associations between quantitative measures of equine lameness/limb abnormalities and reported changes in management and work practices were compared with 21 control (C) communities of owners where no intervention had taken place. Key findings from ‘complete cases’, where the same horse stayed with the same owner for the whole study period (PI group = 73 owners of 83 horses, C group = 58 owners of 66 horses), were that more positive statements of change in equine management and work practices were made by PI group owners than C group owners. A mixed picture of potential risk factors emerged: some reported management improvements, for example reducing the weight of the load for cart animals, were associated with improved limbs and lameness, and others, such as making improvements in shoeing and increasing the age at which their animals started work, with negative outcomes.

**Conclusions/Significance:**

This study illustrates the complexity and interacting nature of risk factors for lameness in working horses, and highlights the importance of longitudinal investigations that recognise and address this. PI group owners found the project useful and requested similar inputs in future. Our findings demonstrate the value of exploratory and participatory research methodology in the field of working horse welfare.

## Introduction

Working horses in low-income developing countries number approximately 17.3 million [1], forming a vital part of urban and rural transport systems and often providing the major or only source of livelihood for their owners. Previous studies have found the prevalence of lameness in working horses to be 90–100% [2,3,4], threatening the income and food security of poor communities and a cause for serious welfare concern.

Animal welfare problems are the result of a complex interaction between layers of causal or risk factors [5]. Welfare outcomes in working equids are a consequence of interactions between animal-related risk factors (age, sex, hereditary and acquired conditions), primary or direct risks related to management of the animal’s living and working conditions (such as feeding, housing and the harnesses and other equipment they are worked in), secondary risks related to compulsions and constraints affecting the owner’s decision-making (knowledge and skills, household income, health service availability, peer pressure and other local factors) and wider socio-economic, cultural and environmental influences.

Risks contributing to lameness and clinical limb abnormalities have been described in dressage horses [6], racehorses [7] and endurance horses [8]; however similar studies relating to working equids are scarce. A higher prevalence of lameness was seen in an urban population of working donkeys in Ethiopia than a rural population [9]; and pack donkeys were found to be more likely to have poor hoof condition and an abnormal gait than draught or ridden donkeys [2]. Environmental and demographic risk factors for poor welfare using data taken from standardised assessments of 5481 donkeys, 4504 horses and 858 mules showed a significantly higher prevalence of tendon and joint abnormalities in urban compared with rural areas, in animals pulling carts compared with other types of work, and in wet seasons compared with dry seasons [4]. However, none of these studies asked horse owners for their opinions on indirect risk factors or ‘root causes’ for lameness, such as the local influences or wider socio-economic conditions that affect their choice of equine training, working or other management practices.

Qualitative research is used in the human health and social science fields to gain insights into ‘real world’ experiences of people, for example affected by disease or social welfare issues. Both qualitative and mixed methods are also widely used in livestock research in developing countries [10], but have been under-utilised in research on working equids. The additional value that qualitative methodology can bring to veterinary research was highlighted by Litva et al [11] who concluded that lay epidemiology is useful for identifying potential causes of animal disease that may be overlooked by conventional veterinary studies, and can uncover barriers to implementation of professional advice. Both of these are highly relevant to the study of lameness in working horses. In addition, qualitative methodologies are valuable in accessing people who are hard to reach using traditional quantitative methods [12]. This is especially true for research in the global south where qualitative approaches typically lead to a greater depth of understanding of local issues than can be ascertained using quantitative tools alone [13].

Quantitative and qualitative methods in field research can combine and complement each other in various ways to improve the trustworthiness of survey and experimental findings [14]. Our current study builds on our previous work [15] by combining quantitative measurements of lameness and limb-related abnormalities with qualitative interview statements describing management changes made by horse owners participating in a 2-year participatory intervention project, in Jaipur, India. Risk factors for lameness were identified retrospectively by identifying statistical differences in welfare and lameness parameters between intervention and control groups of horses, and relating these to reported changes in equine management and work practices, and to any wider socioeconomic and environmental changes over the same period of time.

## Materials and Methods

This study was carried out in and around the city of Jaipur in north-west India between October 2007 and December 2009. It received ethical approval from the University of Bristol (Investigation number UB/07/026) and was compliant with Indian law regarding the ethical use of animals in science.

### Experimental protocol

A participatory intervention (PI) group (21 communities; 248 owners) and a control (C) group (21 communities; 191 owners), matched by locality and the work type of the horses in the community, were identified for this study by staff from the local supporting organisation, Help in Suffering (HIS). The sample was essentially a convenience sample with a matched control group, with the sample size (n = 42 communities) reflecting the number of communities it would be feasible to work with within the timeframe of this study. The study purpose and protocol were verbally explained to participants and informed consent to participate in the study was obtained verbally due to sensitivities regarding the literacy of some participants. The ethical committee gave their approval for this consent procedure. Once consent was obtained the participant was placed on the participant list which was kept confidential. Control groups were only briefed on the monitoring they would receive and not the intervention received by the other group. It should be noted that the PI and C communities were not strictly isolated from each other and social contacts and service providers such as farriers that worked with both community groups were potential contaminants. For further details see [15].

A member of each PI community was trained as a facilitator during a series of training workshops totalling 10 days, prior to the start of the intervention study. First, a range of equine welfare and lameness-related issues were explored using participatory rural appraisal (PRA) exercises [16]. (These are summarised in S1 Supporting Information. Participatory rural appraisal (PRA) exercises used with facilitators). After each training workshop, groups of three facilitators repeated the same exercises with horse-owners in their communities, thus identifying horses’ major welfare needs and potential risks for lameness. These were illustrated on a monitoring (trend analysis) chart in each community. The equine welfare needs identified by PI community facilitators during the initial training period included basic needs such as water, food and protection from the cold; needs related to health such as de-worming, tetanus vaccination, not working during late pregnancy or when sick; and needs relating to their work including their working hours, opportunity to rest, the maintenance and suitability of carts and harnesses, and attention to the weight of loads, speed of travel and the surfaces the horses are worked on.

After their initial training, each facilitator held meetings with horse owners every 1–2 months to discuss equine welfare and management issues; these were often co-facilitated by the project field coordinator (AKD). At each meeting, participants agreed the extent to which each person was providing for his horse’s needs and tracked their progress over time, using coloured stickers or marks on the monitoring chart to represent good, moderate and inadequate actions taken by the owner for each management practice. Over the course of the 2-year project, many participants chose to use the monitoring chart less frequently, although discussions around meeting their animals’ general welfare needs and reducing potential risk factors for lameness continued at regular intervals.

The control (C) group of horse-owning communities did not receive the training, facilitation and meetings described above. Both PI and C groups received intermittent visits from a veterinary mobile clinic run by a local supporting organisation Help in Suffering according to its pre-existing practice. This was completely unrelated to our study.

### Lameness examination

A detailed examination for lameness and musculoskeletal abnormalities (‘lameness examination’) was carried out before the intervention started (Baseline) and after approximately 12 and 24 months (Year 1 and Year 2). The examination contained 41 parameters relating to gait, conformation, feet, limbs and spine, as described in [15,17]. All lameness examinations were carried out by the same clinician (CER) with experience of assessing working equids in the Asian subcontinent.

### Interviews with horse owners

At the Year 2 examination, horse owners were interviewed in their local language, Hindi, to gain retrospective information about any changes in their horses, their equine management practices at home and while working, and the wider environment.

Two interviewers (including AKD) used a 4-part questionnaire, described in Table 1. Responses were translated and recorded in English on the spot. To minimise bias owners were asked to articulate both the positive and the negative changes they had observed over the study duration and then separately to rate the magnitude of the changes they had seen. While it was likely that the responses of owners from both groups were subject to recall bias, for PI group owners this was minimised through their regular meetings over the duration of the study and the use of the monitoring chart to form a diary of changes over this period.

**Table 1:** Components of individual exit interviews with horse owners in participatory intervention (PI) and control (C) groups at the end of the 2-year project.

Interview section	Sub-section and Type	Brief description^1^
Section 1 (all owners)	Semi-structured interview (open-ended questions, qualitative)	What has changed for the horse, in terms of improvements or new problems? Have there been any changes which could have affected the horse: in the household, the horse's work or the policy environment (political, local or national government changes)? Have there been any local initiatives that have helped to improve the lives of horses, owners or the community?
Section 2 (all owners)	Part 2a: Card-sorting exercise (categorical element)	Each horse owner sorted 23 'animal needs' cards^2^ into 3 categories:
		(i) a change which has been positive for the horse (better provision of resource and management needs)
		(ii) no change in meeting the need
		(iii) a change which has been negative for the horse (poorer provision of resource and management needs)
	Part 2b: Card-sorting exercise (quantitative element)	For needs which had shown a change (positive or negative) in Part 2a, each horse owner placed the card at the point on a metre-rule corresponding to the magnitude of change (smallest possible change = 0, largest possible change = 100).
Section 3 (all owners)	Structured lameness questions	Is the horse lame at the moment?
		If so, is it a little bit lame, a moderate amount or very lame?
		If lame, is it working or resting?
Section 4 (PI group owners only)	Semi-structured interview (qualitative)	Has the monitoring chart been beneficial? How has the participatory intervention project affected horse owners and has it been helpful? How could the monitoring chart and the project be changed/ improved for the future?

^1^ Semi-structured interview questions were addressed directly to each horse owner and referred to the 2-year project period only. In some cases the owner was absent and the interview was carried out with a relative.

^2^ Each card stated a single equine resource or management need taken from the monitoring chart used in group meetings, as shown in Table 2. Some cards referred to management or work practices which were only relevant to some horses, for example cart maintenance, feeding during pregnancy. These were omitted from the card-sorting exercise for individual owners if not relevant.

### Qualitative and statistical analysis of interview data

Content analysis of qualitative responses to the open-ended questions (Table 1, Section 1) was carried out according to standard methodology [18] whereby the interview transcripts were systematically reviewed for common themes. Two authors (RMAP & SIH) produced separate lists of themes generated by horse owners in their responses. These themes were not pre-defined but were identified as common topics discussed by horse owners during the interviews. HRW, RMAP & SIH discussed the themes, including any discrepancies between the two lists, and refined them by defining each theme using a collaborative coding process designed to ensure that all owner-generated topics of research interest were represented. This process was aided by the social science software package NVivo 8 (NVivo 8, QSR International Pty, Victoria, Australia). Codes were tested for inter-observer reliability and modified as needed. SIH used the resulting classification system to code the content of all interviews in random order, to reduce the possibility of any intra-observer bias over time being asymmetrically expressed across treatment group or community. The content and structure of the interview meant that it was not possible to code them in a manner which was blind to treatment group assignment. At regular intervals, RMAP coded samples of the interviews to re-check inter-observer reliability: if this fell below criterion, discrepancies were discussed by HRW, RMAP & SIH and resolved by clarifying what each code encompassed and how they differed, with the aim of maintaining a high level of consistency in the coding methodology. Emerging themes were reported qualitatively and also used in quantitative analysis of associations between potential risk factors and lameness or other limb-related outcomes.

The card-sorting element of the exit interview (Table 1, Section 2) was developed from the equine welfare needs identified by PI community facilitators during their initial training, plus management and work practices identified in the root cause analysis and other PRA exercises with groups of owners. In this task, variables ranged from -100 (representing the greatest negative change as measured by the metre rule) to +100 (greatest positive change), with a value of zero assigned when the owner reported no change over the 2-year period.

Interviews were conducted with all participating owners; however, for statistical analysis, as in [15], analyses were conducted with only those data from horses assessed on all three occasions and remaining with the same owner throughout (total n = 131 owners of 149 horses: PI group = 73 owners of 83 horses, C group = 58 owners of 66 horses). To test for any differences, by treatment, in the probability of sorting the needs cards into each “change” pile, a binary variable was created combining “negative change” and “no change” as one category (since there were relatively few interviews indicating the former), with “positive change” as the other. This binary variable was then analysed as the response (y) variable in a separate logistic regression model for each need. Note that when respondents indicated a negative or positive change had occurred, there was relatively little difference, by treatment, in the extent of change as indicated by the metre-rule (i.e. the shape of the distributions were similar for the two treatment groups for each need, and formal testing (when sample sizes were sufficient) confirmed that there were no significant differences by treatment). Random intercept models were constructed with owners nested within community, and treatment added as a fixed effect. Inclusion of community as a random effect controlled for any non-independence at that level. For binary response logistic models, the significance of treatment group (PI or C) as a predictor was assessed using a Wald test and in continuous response models it was assessed via a likelihood ratio test. Ordinal response variables with a relatively large number of categories were usually modelled as a continuous response variable following transformation to Normal scores [19].

An expert panel (HRW, JCP & acknowledged contributor) matched each of the lameness/limb-related outcomes with potential risk factors from the content analysis and card-sorting exercises which could be reasonably hypothesised to have a causal relationship with changes in horse outcomes. The extent of change in each lameness/limb outcome variable across time (Year 2 assessment—Baseline) was analysed as a response variable in statistical models which controlled for treatment group (PI or C), age group of horses and work type (where significant) as fixed effects, with community, animal, and limb added as random effects. Interview-derived variables selected as plausible risks by the panel were added to these models individually to assess the significance and direction of any association.

For discrete response models, the significance of treatment group (PI or C) as a predictor was assessed using a Wald test and in continuous response models it was assessed via a likelihood ratio test. Second order penalised quasilikelihood (PQL) was used as the estimation procedure for discrete response models, and iterative generalised least squares (IGLS) for the continuous response models. Ordinal response variables with a relatively large number of categories were usually modelled as a continuous response variable following transformation to Normal scores. All Analyses were conducted using MLwiN v2.22 [20].

## Results

### Section 1 (open-ended questions): Differences between PI and C groups in their statements of change about their horses, equine management and the wider environment

Table 2 shows the proportion of owners in the PI and C groups making statements of positive and negative change relating to their equine care and working practices, and differences between the two groups. Statements of positive change in general health included:

*“My horses are much healthier than before because of the care that I now take”.*

*“For the last 2 years a change has come in my mare’s health. Mostly this is due to the change in the care that I take”.*

**Table 2:** What changed? Statements of positive and negative changes in equine care over a 2-year period made by participatory intervention (PI) and control (C) groups of horse owners in response to open-ended interview questions at the Year 2 assessment.

Statements of change^1^ in terms of:	Number (%) of respondents out of total n = 131 owners	Number (%) respondents from C group n = 58 owners of 66 horses	Number (%) respondents from PI group of n = 73 owners of 83 horses	Significance of difference between groups (P-value)^2^
*Positive changes*				
Diet	120 (53)	32 (31)	88 (72)	<0.0005
Cleanliness of food	28 (12)	0	28 (23)	<0.0005Ψ
Water provision	21 (9)	0	21 (17)	<0.0005Ψ
Health	82 (36)	34 (32)	48 (39)	0.295
General care	45 (20)	6 (6)	39 (32)	<0.0005
Shoeing	39 (17)	7 (7)	32 (26)	<0.0005
Hoof care	14 (6)	3 (3)	11 (9)	0.094Ψ
Protection from weather	15 (7)	4 (4)	11 (9)	0.179Ψ
Grooming, bathing and cleaning	31 (14)	3 (3)	28 (23)	<0.0005Ψ
Harnesses, bits and saddles	37 (16)	13 (12)	24 (20)	0.138
Cart and loading procedures	7 (3)	1 (1)	6 (5)	0.127Ψ
Working hours	11 (5)	0	11 (9)	0.001Ψ
Riding and handling	52 (23)	14 (13)	38 (31)	0.001
Selection of routes	21 (9)	0	21 (17)	<0.0005Ψ
Rest	6 (3)	0	6 (5)	0.032Ψ
Exercise	3 (1)	0	3 (2)	0.251Ψ
Owner Knowledge	75 (33)	1 (1)	74 (60)	<0.0005
Owner attitude	6 (3)	0	6 (5)	0.032Ψ
Lameness	11 (5)	1 (1)	10 (8)	0.012Ψ
*Negative changes*				
Diet	31 (14)	18 (17)	13(11)	0.153
Health	20 (9)	13(12)	7(6)	0.081
Shoeing	11 (5)	5(5)	6(5)	0.967
Lameness	12 (5)	5(5)	7 (6)	0.753

^1^ Statements of change represent major themes extracted from content analysis of exit interviews with participating horse owners (see Table 1) using NVivo software.

^2^ P values from a test of 2 binomial proportions, comparing the C and PI groups.

^Ψ^ = P values from a Fisher's exact test (where normal approximation may be inaccurate).

An improvement in the incidence of colic was a particularly noticeable theme, with 14 PI group owners (11%) compared to 2 C group owners (2%) stating that colic occurred less often or not at all now.

*“Earlier I was careless in feeding my animal. Used to throw food and because of that dust and dirt used to go into its stomach. Now I clean the food first, so no colic problem now”.*

*“Colic was more prevalent 2 years back. Now I add salt and oil regularly, so the stomach of my horses is much better now”.*

*“The main thing that happened through this work was—my horse never again suffered from colic. It has started eating well. Looks energetic always”.*

Eleven owners made statements of a positive improvement in their animals’ limb abnormalities or lameness; 10 of these were from the PI group. Two PI owners stated that lameness had improved due to advice from the project. Other specific reasons mentioned were:

*“Started checking the swelling in the feet after those monitoring chart meetings”.*

*“Also because of less rains the good thing that has happened is that the hoof of the horse has become strong as it didn’t get wet”.*

*“There was injury at the hind leg of one of my mare which happened due to an accident 1 ½ years back since then I am taking extra care whenever I ride it. I ride the horses slowly now”.*

PI group owners’ statements about general hoof care and grooming included:

*“Earlier the hoofs were more dirty. After all those meetings I started checking them before I take them for work”.*

*“Me and my brother take care that our horses don’t stand in moist places for longer time. It spoils the hoofs”.*

*“Earlier no horse owner bothered to examine the hoofs of horses, now everyone does it. I now take more care of hoofs and shoeing”.*

In the PI group 26% of all owners made statements of a positive change in shoeing compared to 7% of the C group. Reasons for improvement in the PI group included: shoeing had become more ‘timely’; the farrier’s work had improved; the owner had changed to a better farrier or started to shoe horses himself; the owner cleans, measures or levels the hoof first or instructs the farrier to do so; the owner uses better shoes; he gets all 4 feet shod at the same time. Different reasons were given by C group owners for improved farriery: they now have fewer horses to look after, they clean the hoof first, they now purchase shoes directly from the farrier rather than buying them somewhere else and the owner now goes to the farrier himself rather than ‘sending his kids’.

Eleven PI group owners (9%) said they had reduced the working hours or demands made on their horses; no C group owners mentioned this. Most simply stated that they had reduced their working hours, although 2 owners said they had reduced the number of passengers they take. Two owners said they take their horse to work in a truck if they need to travel a long distance, rather than making it walk, and 1 owner said he does not use his pregnant mare for work any more. Thirty-one percent of PI owners made statements of positive change in the ‘riding and handling’ of their animals compared to 13% of the control group. Fourteen percent of the PI group specifically mentioned a reduction in driving speed compared to only 1% of the control group. A further 2 PI group owners knew that it was important not to drive fast but did not confirm that they had put this into practice. Statements of a positive change in the selection of the routes they used for work were made by 17% of the PI group; no C group owners mentioned this. A further 6 PI owners (5%) stated that they knew route selection was important but did not confirm that they had made a change in practice.

*“He has observed some difference in general practices that they have stopped like—going through rough tracks and riding too fast”.*

*“I started riding with slow speed. I started providing rest and letting it roll over whenever it wanted”.*

*“I personally took care of all things you explained. Fed it well, and drove it well. Drove it slow. I stopped giving sudden brakes, rode it slow”.*

*“Started riding carefully protecting horse from rough surface and obstacles”.*

*“I now take good care so much as I even take care that its foot doesn’t fall in faeces”.*

Seven owners (6 PI, 1 C) said that they had improved their care of the cart or the loading procedures. Three PI group owners reported that they had increased cart maintenance or made it more ‘timely’, 2 said they had improved the cart and equipment and 1 said he had reduced the load carried on the cart. The C group owner said he now had fewer horses and more time to concentrate on cart maintenance.

Only one C group owner mentioned an increase in his knowledge over the 2-year duration of the project, which he stated was “gained by talking to others and seeking information”. In contrast, 60% of PI owners made comments concerning the increase in their knowledge during the project, many of them mentioning the specific nature of this knowledge. Quotes from members of the PI group included:

*“Yes it was helpful to me. It increased my knowledge about horses and I was more responsible towards my horses after this work”.*

*“I learnt that if we take good care of the animal then we can get more customers and earn well”.*

*“Due to the understanding developed amongst him and his family about his horse, they started taking more care of their animal and they started loving it more than before”.*

*“I learnt and applied things like: to ride the animal slowly, to take it through proper tracks, to add salt to the diet and to love and groom the animal daily. My mare got healthier than before”.*

### Section 2 (card-sorting exercise): Differences between PI and C groups in the type and extent of reported changes in equine management over the 2-year project

Table 3 illustrates statements of positive and negative change in equine management practices and lameness which differed significantly between owners belonging to PI and C groups.

**Table 3:** To what extent did it change? Direction, extent and differences in reported changes in equine care over a 2-year period, made by participatory intervention (PI) and control (C) groups of owners in response to a card-sorting exercise.^1^

				Extent of reported change (-100 = greatest negative change; 0 = no change; +100 = greatest positive change)							
		Number of interviews^2^		Overall		Control (C)		Participatory intervention (PI)		Difference in mean between groups (PI-C)^4^	
Category	Equine welfare need	C	PI	Mean	(SD)	Mean	(SD)	Mean	(SD)		P-value^3^
Diet	Water	105	123	25	(35)	17	(30)	32	(37)	15	0.027
	Food	105	123	27	(39)	12	(37)	39	(36)	26	<0.001
	Milk	102	120	8	(29)	7	(32)	9	(27)	1	0.836
Work	Working hours	96	106	9	(28)	9	(27)	9	(28)	0	0.346
	Rest during day	105	123	23	(36)	19	(36)	26	(36)	7	0.442
	Weight of load	27	38	9	(23)	7	(25)	10	(22)	3	0.272
	Working on hard/uneven surfaces	105	118	33	(38)	20	(36)	45	(37)	26	<0.001
	Driving speed	100	120	27	(36)	18	(35)	35	(35)	17	<0.001
Shoes and equipment^5^	Shoes	103	119	21	(42)	9	(39)	32	(41)	23	<0.001
	Harness	104	121	27	(45)	15	(49)	37	(39)	22	0.052
	Cart maintenance	29	34	8	(35)	-2	(40)	16	(28)	17	0.114
Rest when pregnant or sick	Not working in late pregnancy	91	105	18	(33)	9	(25)	25	(38)	15	<0.001
	Working while sick	93	103	6	(23)	6	(24)	5	(21)	-1	0.635
Early life	Age starting work	91	101	22	(35)	12	(29)	30	(38)	18	0.003
	Care of newborn foal	91	103	24	(35)	19	(33)	27	(36)	8	0.133
Protection from elements	Stabling	104	123	29	(37)	21	(36)	36	(37)	15	0.012
	Protection from cold	105	123	21	(35)	18	(34)	23	(36)	6	0.276
	Hair clipping	104	121	8	(25)	7	(23)	9	(26)	2	0.684
Misc. good practices	Massage	105	123	27	(43)	12	(41)	40	(40)	27	<0.001
Misc. poor practices	Tethering (tying of legs)	103	120	7	(31)	-1	(26)	13	(33)	14	0.007
	Dancing ^6^	85	104	7	(32)	-2	(27)	15	(34)	17	0.002
Preventive veterinary care	De-worming	102	123	26	(38)	7	(29)	41	(37)	34	<0.001
	Tetanus vaccination	102	123	27	(39)	7	(32)	43	(37)	35	<0.001

^1^ See Table 3 for details:

^2^ Excluding 'not applicable' questions and missing responses:

^3^ Significance of difference between PI and C groups in the probability of owners indicating positive change (compared to negative or no change):

^4^ A positive number indicates that the participatory intervention group reported a greater improvement in meeting their horses' welfare needs than the control group; a negative number indicates the opposite:

^5^ A positive change in shoeing may indicate more frequent shoeing or a perceived better quality service; a positive change in harness may indicate either a better harness or better care of the existing harness:

^6^ Many horses were trained to carry out piaffe movements for prolonged periods to entertain onlookers at ceremonial events; agreed by facilitators to have negative physical and psychological effects on welfare.

The most significant differences between PI and C groups occurred in response to questions about: diet, cleanliness of food and water provision; general care including grooming, bathing and cleaning; lameness; shoeing; handling, riding/ driving, working hours and route selection. There was a general tendency, across both treatment groups, to indicate that positive change or no change (rather than negative change) had occurred in the extent to which animal needs were met. The PI group responded that positive change occurred more frequently than did the C group (LRT statistic = 21.01; p<0.001), whilst the C group indicated that no change (LRT statistic = 20.96, p<0.001) or a negative change (LRT statistic = 7.65, p = 0.006) occurred more frequently than the PI group. However, when respondents indicated that a change (negative or positive) had occurred, there was relatively little difference between PI and C groups in the extent of that change.

### Section 3: Associations between management changes described in the interviews and animal-based outcome measures of lameness and limb abnormalities

Significant relationships between stated management changes and lameness or limb outcomes across both groups are reported in Table 4.

**Table 4:** Significant associations between the change in lameness/ limb-related horse outcome measures (Year 2 compared with Baseline) and management changes over the 2-year project (as reported at the Year 2 owner interviews).

Management change over the 2-year project^1^	Lameness/ limb outcome^2^ ^,^ ^3^	Wald statistic	P value
Less work on hard uneven surfaces	Reduced FL fetlock angle (varus/ valgus)	8.17	0.004
	Worse FL hoof conformation	6.96	0.008
	Worse FL frog quality	6.57	0.010
	Worse HL full limb flexion range of movement	7.71	0.005
Less weight of load (cart animals only)	Less FL pain response to hoof testers on craniomedial sole	5.92	0.015
	More improvement or less deterioration in overall lameness score	5.80	0.016
Fewer working hours	More FL pain response to hoof testers on any area of sole	5.30	0.021
	More pain response to HL full limb flexion	11.95	0.001
Slower riding/ driving speed	More FL pain response to hoof tester on any area of sole	4.02	0.045
	Less likely to have lesions at commissures of lips	7.70	0.006
Increased age starting work	More HL pain response to hoof testers on any area of sole	5.44	0.020
	Less improvement or more deterioration in HL lameness score	8.00	0.005
	Less likely to have lesions at commissures of lips	6.83	0.009
Less working while sick	More FL pain response to hoof testers on craniolateral sole	3.94	0.047
	Worse hoof conformation	5.52	0.019
More rest during day	Less FL pain response on any area of sole	4.89	0.027
Less dancing	Less HL pain response to hoof testers on centre of frog	4.33	0.037
Better shoeing/ farriery	Less improvement or more deterioration in overall lameness score	3.85	0.050
	Less improvement or more deterioration in HL lameness score	11.23	0.001
	Improved HL hoof-pastern axis	5.52	0.019
	Less concave HL sole structure	3.96	0.047
	Less likely to be shod	4.24	0.039
	Better HL shoe quality	9.03	0.003
Better harness	Less improvement or more deterioration in HL lameness score	10.26	0.001
Better tetanus vaccination practices	More improvement or less deterioration in overall lameness score	4.70	0.030
More knowledge	Less improvement or more deterioration in overall lameness score	9.55	0.002
	Less improvement or more deterioration in HL lameness score	6.42	0.011
Better general care	Better FL frog quality	9.23	0.002

^1^ All derived from card-sorting exercise (Table 1 Section 2) except * = derived from content analysis (qualitative assessment) of responses to open-ended questions (Table 1 Section 1).

^2^ Changes in limb outcome are described for owners who reported improvement in the management/work practice over 2 years, as compared to owners who reported making no change or a deteriorated management/work practice.

^3^ FL = forelimbs, HL = hindlimbs

### Section 4: Opinions of the PI group on the participatory intervention project

Sixteen PI owners (13%) who attended the interview indicated that they had not been to any of the meetings or did not know about the project; 11 of these were taking part in the interview on behalf of someone else. A further 16 owners (13%) knew about the project but had not been able to attend many meetings for various reasons, including the distances involved, lack of information about meeting dates and lack of time to attend.

The equine management monitoring chart was introduced to PI owners during their initial training session and so even if an owner had been unable to attend the meetings they were aware of the chart, its contents and meaning. During the interview the equine management monitoring chart was mentioned by 98% (121) of PI owners and 99 of these were very positive about it. Comments included:

*“Yes it helped in improving the condition of legs and other well being of animal.”*

*“The two things that strike him in the monitoring chart are that the proper cleanliness around the horse is maintained, it prevents infections and diseases.”*

*“Yes, it was very helpful. I learnt many things about my animal and general care like—feeding, riding, cleanliness.”*

From the statements it is not known how many owners had actually put into practice what they had learnt from the monitoring chart, although 11% of owners did go on to confirm that they had made changes in the care of their horses using the information gained from the chart.

PI group horse owners liked the visual nature of the monitoring chart, with one saying it was good even for illiterate people and another that it would be particularly useful for new horse owners who know very little about horses. In response to a question about improving the chart, most owners had “no idea”, but several suggested that the horse owners themselves should get together to discuss it. Four owners felt that the chart should contain information about medicines, including their action and doses, while other suggestions are quoted below:

*“Probably there should be more welfare needs mentioning/ highlighting the bone deformities and how to handle them to relieve the animal”.*

*“Certain local healthy diets like—gram seeds, pulses along with milk etc. should be included in that chart. Such things that make the horse more powerful”.*

When asked to assess the project overall, 87% of PI owners made comments and most of these were positive, with owners stating that the project: was good/ effective /well run; had a good mode of instruction; was informative/helpful /increased their knowledge; taught them the do’s and don’ts; provided relevant information; was easy to understand; was enjoyable/interesting; changed or affected their behaviour; was good for new owners; was good because owners could discuss things together; and reminded them of things they had forgotten. There were few negative comments: that the project only benefited the facilitators who were paid to attend the training; that ‘careless’ people don’t attend the meeting; that the veterinary treatment vehicle (run independently from the lameness project) did not attend regularly, and:

*“The work is good but these days no one has time for such work”.*

A control group owner also made a negative comment about the project:

*“You should think about it. You have been examining our horses for the last 2–3 years but you only check them and don’t give us any results or medicine.”*

Suggested improvements to the project included that the meetings should continue or even increase; however the main focus for specific improvements were that more medicines or a medicine box should be provided and that the veterinarian should visit more often. Five owners suggested that the improvements should in fact come from them; they should take more care, invite family members, listen and cooperate.

## Discussion

In this study, quantitative and qualitative methods were used to compare owners’ reported changes in equine management and work practices over the 2-year project lifetime with changes in their horses’ lameness and other limb-related outcomes measured on 3 occasions over the same period. The inductive approach, generating qualitative information via a variety of techniques, is common in social science research and increasingly in medical contexts [21,22]. In particular it has the purpose of understanding rather than measuring phenomena, and conveys richness of detail and meaning which is lost when reducing information to discrete variables and linear cause-effect relationships [23]. To the best of our knowledge, this is the first longitudinal study using mixed methods analysis to attempt to identify risk factors for lameness and limb problems in working horses. Key findings were: that more positive statements of change in equine management and work practices were made by horse owners in the participatory intervention (PI) group than the control (C) group; that a mixed picture of potential risk factors emerged, with some reported management improvements associated with improved limb and lameness outcomes, and others associated with negative outcomes; and that owners taking part in the intervention found it useful and requested similar inputs in the future.

In general, PI owners were more likely to report positive change in the management and work practices identified by qualitative content analysis of the open-ended interview questions and quantitative analysis of the card-sorting exercise. Some of these, such as reducing the speed of work and avoiding rough and hard ground, were attributed directly to the intervention project. Others were attributed to external influences such as less rain, or an accident involving the horse, and some owners did not state why they had made a decision to change their practices. This concurs with the categories or layers of risk discussed previously [5]: an animal’s welfare is determined not only by the action or inaction of its owner or user, but also by wider social, cultural and environmental factors influencing their decision-making processes and directly influencing the animal itself. However, the possibility that PI group owners may have responded more positively than C group owners to encourage further funding or charity involvement in their area should also be considered.

Some of the positive changes reported were directly related to lameness and limb problems, notably shoeing, driving/ riding speed and selection of better routes or terrain. Many were not; in particular the largest number of positive management changes in the PI group, and second largest in the C group, were related to diet. Improved cleanliness of the feed, offering salt and oil with the feed, better watering arrangements and a reduced incidence of colic were mentioned frequently in both qualitative and quantitative elements of the PI group exit interviews. This may reflect a particular interest in colic risk reduction among horse owners, possibly due to its dramatic presentation and consequences in comparison with limb-related issues. It may also be due to a preference or emphasis by the community facilitator when discussing this topic at group meetings, or because diet-related changes are relatively easy to make within horse owners’ budgetary and other constraints; changes in diet may also produce immediate positive results, whereas lameness problems may be chronic and irreversible in some cases. Fewest positive responses were received relating to reducing working hours, increasing rest periods and reducing workloads, and the extent to which these factors were reported to have changed did not differ significantly between PI and C groups. Although they did not appear as statements of negative change or deterioration over the two year project, it may be that these work practices are some of the hardest welfare risk factors for owners to influence in a positive direction because they are in direct conflict with the need to earn a living from working animals.

Improved harness and cart maintenance during the project period were mentioned by some PI owners during the open-ended interview, although the extent of change did not differ significantly between PI and C groups in the card-sorting exercise. Reasons for discrepancy between qualitative and quantitative reporting could be that when giving numerical ratings or scores to behaviour change questions, respondents are likely to answer quickly and may include their perception of the correct answer or societal norm. Although this type of bias can also occur in answers to open-ended questions, respondents may be more likely to discuss their general priorities or give the answer that is uppermost in their mind at the time of questioning, and further probing or discussion may result in a more considered answer. Either method may be considered to give a more objective result, depending on the criteria used for objectivity.

The quantitative element of the exit interview produced a much higher response rate than the qualitative element, especially in C groups where qualitative responses were low. It ‘forced’ an answer from each interviewee, whereas the open-ended questions may or may not elicit responses across the full range of equine management changes addressed by the project. For example, in the card-sorting exercise PI owners reported having improved their practices relating to limb-tethering significantly more than C group owners, yet this did not emerge as a theme from the content analysis of their interviews, perhaps because they did not consider it to be one of the most important or interesting management changes they had made. For this reason quantitative evaluation may be seen as more useful when assessing projects of this type. However, in agreement with qualitative researchers in the medical field [23], the benefits of thematic content analysis included diverse, rich and colourful accounts of respondents’ lives, including the constraints faced when attempting to meet their animals’ needs in the face of environmental challenges beyond their control and competing priorities for income generation and expenditure. Qualitative investigation enabled a better understanding of the whole complex picture of the working lives of horses in this region.

Quantitative statistical analysis of relationships between the extent of change in lameness/ limb-related outcomes and horse owners’ reported management changes yielded mixed results, some of which were counter-intuitive. For example, there was a more positive change in lameness (more improvement or less deterioration) in cart horses whose owners reported reducing the load carried or improving the tetanus vaccination status, compared with horses whose owners did not make these changes. The first of these seems to be a logical outcome through less musculoskeletal strain; the second does not appear to indicate a causal relationship although may be correlated through a general increase in care and attention to animal needs. A greater negative change in lameness (less improvement or more deterioration) was seen in horses whose owners reported making improvements in shoeing and increasing the age at which their animals started to work. The former was unexpected and may be due to poor shoeing techniques in practice, despite the owner perceiving it to have improved; the latter may also be counter-intuitive, although a similar finding has been reported in racehorses where early conditioning was associated with no adverse effects and some positive effects during later exercise [24]. Pain responses to frog pressure showed some improvement where dancing (piaffe) was reduced or stopped, but increased sole pain was seen in animals whose owners reported doing less work on hard, uneven surfaces, and not all areas of the sole were affected in the same way by reported management changes. The reduction in bit-related lip lesions and scars in horses that had started work at an older age and in those whose owners reduced their riding/driving speeds was an encouraging sign that improved work practices could make a difference to this particular issue.

As previously described, risks to welfare are complex and interact with each other at several levels, so it was always unlikely that a set of linear cause-and-effect relationships would emerge from this study. The management changes identified by community facilitators as potential risk factors for lameness and limb-related problems may not be the right ones to effect a positive change: as found in other studies, lay theories may or may not be in agreement with professional ones [11,25]. Owners may have made different choices or interpretations of the actions needed to address their horses’ needs and reduce lameness risks. Regardless of their efficacy, the management changes discussed during group sessions may not have been acted on by all owners, for many reasons including individual cost/benefit considerations. However, the negative management changes reported in relation to diet, health, lameness and shoeing during the exit interviews did not differ significantly between PI and C groups of horse owners, suggesting that the project intervention did not inadvertently encourage changes for the worse.

Most owners from the PI group were very positive about the intervention project and wished for it to continue. Many suggestions for improvement were focused on fluctuations in the frequency and perceived quality of free veterinary treatment being provided in parallel with, but not linked to, the lameness intervention. Ideally the two interventions would have worked in close collaboration and this illustrates the challenge of disentangling elements of causality or attribution within the study. However, it also reflects the reality of the circumstances in many developing countries, where several agencies or projects may be influencing horse owners and/or their animals at any one time, sometimes with competing demands. The participatory nature of the lameness intervention aimed to increase owners’ sense of empowerment to influence the well-being of their animals. The ethics of using a control group was identified by the C group owner who commented that the project kept assessing his horses but not helping him, and this is an area of ongoing concern when designing any kind of field research with disadvantaged people [26].

## Conclusion

Despite the absence of a clear picture of risk factors for lameness and limb-related problems in working horses, this study illustrated the importance of longitudinal exploratory investigations that recognise and attempt to address complexity and dynamic welfare change through mixed methods analysis. It could be argued that the study design should have constrained variability and reduced experimental noise; however it would not then have achieved its main purpose, which was to understand the ‘real world’ conditions in which working horses must function. Underestimating complexity at the research and planning stages of an intervention frequently leads to failed implementation of the resulting recommendations, because these are unrealistic and do not take sufficient account of animal owners’ opinions and dynamic or limiting factors within the system and environment [27]. Attempting to impose particular activities or ‘solutions’ on horse owners may have understandably met with resistance or non-adoption. Without exploratory and participatory methodology it would not be possible to decide which risks are most important to investigate further, which are more or less amenable to different change efforts and which research methods to use in the future. Qualitative research findings always raise further questions and the nature of these will dictate the research approach needed for the next study [28]. In the current investigation, learning about the research process and the most appropriate questions to ask were as important as identifying specific outcomes. Clearer risk factors for lameness may have emerged if owners’ management and work practice changes had been reported and measured (where feasible) at more frequent intervals, including recording at baseline, and this will be taken into account for future studies of working animal welfare.

## Supporting Information

S1 Supporting Information Participatory rural appraisal (PRA) exercises used with facilitators. (DOCX)

## References

[1] FAOSTAT Food and Agricultural Organisation Statistical Database, Live Animals. 2010. Available: http://faostat.fao.org/site/573/DesktopDefault.aspx?PageID=573#ancor. Accessed: 2011 Apr 8.

[2] Pritchard JC, Lindberg AC, Main DCJ, Whay HR. Assessment of the welfare of working horses, mules and donkeys, using health and behaviour parameters. Prev. Vet. Med. 2005; 69, 265–283. 15907574 10.1016/j.prevetmed.2005.02.002

[3] Broster CE, Burn CC, Barr ARS, Whay HR. The range and prevalence of pathological abnormalities associated with lameness in working horses from developing countries. Equine vet. J. 2009; 41(5), 474–481. 19642408 10.2746/042516409x373907

[4] Burn CC, Dennison TL, Whay HR. Environmental and demographic risk factors for poor welfare in working horses, donkeys and mules in developing countries. Vet. J. 2010; 186, 385–392. 10.1016/j.tvjl.2009.09.016 19926316

[5] Whay HR. The journey to animal welfare improvement. Animal Welfare 2007; 16(2), 117–122.

[6] Murray RC, Walters JM, Snart H, Dyson SJ, Parkin TDH. Identification of risk factors for lameness in dressage horses. Vet. J. 2010; 184(1) 27–36. 10.1016/j.tvjl.2009.03.020 19369100

[7] Williams RB, Harkins LS, Hammond CJ, Wood JLN. Racehorse injuries, clinical problems and fatalities recorded on British racecourses from flat racing and National Hunt racing during 1996, 1997 and 1998. Equine vet. J. 2001; 33(5), 478–486. 11558743 10.2746/042516401776254808

[8] Nagy A, Murray JK, Dyson S. Elimination from elite endurance rides in nine countries: a preliminary study. Equine vet. J. 2010; 42(Suppl. 38), 637–643. 10.1111/j.2042-3306.2010.00220.x 21059073

[9] Morgan R. The epidemiology of lameness in working donkeys in Addis Ababa and the central Oromia region of Ethiopia: a comparative study of urban and rural donkey populations. Proceedings of the 5^th^ International Colloquium on Working Equids, Addis Ababa, Ethiopia, 30^th^ October— 2^nd^ November 2006, pp99–106.

[10] Conroy C. Participatory livestock research: a guide. Rugby: ITDG Publishing; 2005 pp 58–59.

[11] Litva A, Robinson CS, Archer DC. Exploring lay perceptions of the causes of crib-biting/ windsucking behaviour in horses. Equine vet J. 2010; 42(4) 288–293. 10.1111/j.2042-3306.2009.00025.x 20525045

[12] Christley RM, Perkins E. Researching hard to reach areas of knowledge: Qualitative research in veterinary science. Equine vet J. 2010; 42(4) 285–286. 10.1111/j.2042-3306.2010.00074.x 20525043

[13] Upjohn MM, Attwood GA, Lerotholi T, Pfeiffer DU, Verheyena KLP. Quantitative versus qualitative approaches: A comparison of two research methods applied to identification of key health issues for working horses in Lesotho. Prev Vet Med 2013; 108: 313–320. 10.1016/j.prevetmed.2012.11.008 23419786

[14] Marsland N, Wilson I, Abeyasekera S, Kleih U. Combining quantitative (formal) and qualitative (informal) survey methods. Socioeconomic Methodologies for Natural Resources Research Best Practice Guidelines. Chatham: Natural Resources Institute; 2001.

[15] Reix CE, Dikshit AK, Hockenhull J, Parker RMA, Banerjee A, Burn CC, et al A two-year participatory intervention project with owners to reduce lameness and limb abnormalities in working horses in Jaipur, India. PLOS ONE In press. 10.1371/journal.pone.0124342 PMC4405470 25898014

[16] Kumar S. Methods for community participation: a complete guide for practitioners. Rugby: Intermediate Technology Publications Limited; 2002.

[17] Broster CE, Burn CC, Barr ARS, Whay HR. Prioritising indicators of lameness and related pain in working equids to be included in a practical field lameness assessment tool. Proceedings of the 6th International Colloquium on Working Equids, New Delhi, India, 29^th^ November— 2^nd^ December 2010, pp 9–11.

[18] Bazeley P. Qualitative Data Analysis with NVivo. London: Sage Publications; 2007.

[19] Rasbash J, Steele F, Browne WJ, Goldstein H. A User’s Guide to MLwiN, v2.10. University of Bristol: Centre for Multilevel Modelling; 2009.

[20] Rasbash J, Browne WJ, Healy M, Cameron B, Charlton C. MLwiN Version 2.22. University of Bristol: Centre for Multilevel Modelling; 2010.

[21] Babbie ER. The practice of social research. California: Thomson Wadworth; 2007 pp 44–54.

[22] Britten N. Qualitative research on health communication: What can it contribute? Patient Education and Counselling 2011; 82, 384–388. 10.1016/j.pec.2010.12.021 21242048

[23] Forman J, Creswell JW, Damschroder L, Kowalski CP, Krein SL. Qualitative research methods: key features and insights gained from use in infection prevention research. Am. J. Infect. Control 2008; 36, 764–71. 10.1016/j.ajic.2008.03.010 18834752

[24] Rogers CW, Firth EC, McIlwraith CW, Barneveld A, Goodship AE, Kawcak CE, et al Evaluation of a new strategy to modulate skeletal development in racehorses by imposing track-based exercise during growth: The effects on 2- and 3-year-old racing careers. Equine vet. J. 2008; 40(2) 119–127. 18093893 10.2746/042516408X266088

[25] Frankel S, Davison C, Davey Smith G. Lay epidemiology and the rationality of responses to health education. Br. J. Gen. Pract. 1991; 41, 428–430. 1777300 PMC1371828

[26] Whay HR. The ethics of conducting animal welfare research in poor communities. The 6^th^ International Colloquium on working Equids, New Delhi, India, 29^th^ November— 2^nd^ December 2010, pp 67–69.

[27] Jones H. Taking responsibility for complexity: how implementation can achieve results in the face of complex problems Working Paper 330. London: Overseas Development Institute; 2011.

[28] Wuest J. Are we there yet? Positioning qualitative research differently. Qual Health Res 2011; 21(7), 875–883. 10.1177/1049732311401424 21368018

