# Supplementary material for: Evaluation of Changes in Equine Care and Limb-Related Abnormalities in Working Horses in Jaipur, India, as Part of a Two Year Participatory Intervention Study
Source: PLoS One. 2015 May 22;10(5):e0126160. doi: 10.1371/journal.pone.0126160 (PMC4441452; doi:10.1371/journal.pone.0126160)
Supplement: S1 Supporting Information — (DOCX) [file pone.0126160.s001.docx]

S1 Supporting information. Participatory rural appraisal (PRA) exercises used with facilitators

| PRA exercise | Purpose (identifying, discussing/analysing, scoring) |
| --- | --- |
| Root cause analysis for lameness | Key causes for lameness, relevant to type of work carried out by horses in the community. |
| Well-being analysis | How well- or badly-off horse owners are, compared to other people in the locality; criteria for well-being. |
| Horse-owning livelihoods analysis | How horse-owning livelihoods compare with other livelihoods, on the basis of returns, year-round work availability and hardships involved. |
| Seasonality diagram | Seasonality of income and expenditure, dependence on credit, equine diseases, fodder and water availability. |
| Expenditure analysis | Sources of expenditure including those related to horse maintenance; trade-offs between different sources of expenditure. |
| Credit analysis | Sources of credit according to frequency of use, favourability of terms, availability, choice and size of loan available. |
| Analysis of equine management practices | Good/ appropriate equine management practices, their current prevalence and potential effects on lameness when not carried out. |
| Sources of knowledge | Sources of knowledge/ information on horse breeding, purchasing, farriery and lameness. |
| Flow diagram of equine work | Stages or activities during a horse's working day; roles played by men, women and children at each stage; stages that could contribute to lameness. |
| Progression of lameness | Developing a lameness scoring system, including number of scores and criteria for each score. |
| Welfare needs of horses | Welfare needs of horses, for regular scoring and monitoring. |
| Analysis of key service providers | Key service providers needed for care and maintenance of horses and equipment, including scoring according to accessibility, cost and quality. |
| Impact of demands of employers or clients | Unreasonable demands of customers, clients or employers; how commonly these are encountered; identifying which demands are unreasonable to horses as well as owners. |
| Planning of next steps | Key issues arising from exercises above; prioritising issues; action planning. |
|  |  |
